# Biomodulina T (InmunyVital^®^) Restores T Cells and Helps Contain COVID-19

**DOI:** 10.3389/fimmu.2020.606447

**Published:** 2020-12-09

**Authors:** Roman R. Rodriguez Martin, Olivia Gonzalez Gonzalez, Carolina Rodriguez Gonzalez, Rene R. Rodriguez Gonzalez

**Affiliations:** BIOLONG Corporation, Miami, FL, United States

**Keywords:** Biomodulina T, InmunyVital, T cells, COVID-19, immunomodulator, immunotherapy, vaccine enhancer, immunosenescence

## Introduction

T-cell levels and their functions are related to the severity and lethality of COVID-19. T-cells develop in the thymus gland and play a central role in the adaptive immune response. During aging, the thymus gland slowly shrinks, and levels of the thymic factors it produces decrease in the blood, reducing T-cell differentiation. Moreover, thymus-dependent immunity, including T-cell immunity and thymus-dependent antibody production, diminishes with thymus size in adults, which is known as “immunosenescence.” This is the main reason older adults and immunocompromised individuals are generally at a higher risk of becoming severely ill from COVID-19.

## Discovery and Development of Biomodulina T

In 1984, with an early vision of the importance of obtaining biological response modifiers for use in clinical medicine to fight aging-related diseases, cancer, and other diseases, I created and founded a new laboratory that I named “Laboratorio de Biomoduladores” (Biomodulators Laboratory) in Havana city. In this laboratory, I discovered and led the development of a thymus factor that has an immunomodulatory effect. I named this thymic factor “Biomodulina T” (BMT) due to its main action as a biomodulator, and the letter T was chosen due to the origin of these factors, namely, the thymus gland, and the fundamental action of these factors in stimulating T lymphocytes (1–6).

## Biological Activity of Biomodulina T

We evaluated the immunomodulatory effect of Biomodulina T in Active T Rosette Bioassay. This test identifies a subpopulation of T lymphocytes with a high-avidity receptor for sheep red blood cells and is related to the lymphocyte stimulation test. Neoformation of active T rosettes is due to the early release of a differentiation factor. In this test, we used lymphocytes obtained from the blood of a patient with T-cell immune deficiency and ataxia-telangiectasia. Immune deficiency in ataxia-telangiectasia (A-T) has been suggested to be a result of premature aging of the immune system. This immune aging occurs in the absence of previous infection with cytomegalovirus (CMV), which is the environmental factor most strongly associated with immune aging. The deficiency of naïve T-cells in A-T is similar to the effects of early thymectomy. The pattern of immunodeficiency in classical A-T is typical of aging, with lymphopenia affecting CD4+ and CD8+ T-cells and B cells. There is also a relative deficiency of classical naïve CD4+ CD45RA+, thymic naïve CD4+ CD31/45RA+ and naïve CD8+ CD27/28/45RA+ T-cells and naïve CD19+ B cells. Thus, immunodeficiency in A-T particularly affects naïve T-cells and B cells, which is a typical feature of aging (7).

Biomodulina T exhibited biological activity in this *in vitro* assay, stimulating rosette formation by lymphocytes bound to sheep erythrocytes. Using this assay, we found that Biomodulina T increased the number of T-cells. The newly formed active T rosettes are an early marker of T-cell activation. The increase in the percentage of active T rosettes is related to the ability of Biomodulina T to modulate T-cell functions (6, 8).

Rosette test *in vitro*. A proportion of guinea pig lymphoid cells have an affinity for rabbit erythrocytes, which is demonstrable by a rosette-forming technique. This affinity for rabbit erythrocytes represents a marker of thymus-derived T lymphocytes in guinea pigs in the same way that rosette formation with sheep erythrocytes represents a marker of T-cells from human lymphocytes (9). Biomodulina T exhibited biological activity in thymocytes from guinea pigs, stimulating expression of the receptors specific for rabbit erythrocytes that were previously depleted by trypsin (6, 10).

Rosette test *in vivo.* Biomodulina T increased the ability of guinea pig thymocytes to form rosettes 72, 48, and 24 h after administration. This effect was greater in the group treated with Biomodulina T 24 h before carrying out the assay. Biomodulina T exerts a protective effect against the action of the enzyme trypsin (6, 10).

Lymphocyte blast transformation test. Biomodulina T increased the response to PHA in low-responding individuals but did not increase that in normal-responding individuals. Biomodulina T acts as a regulator of the immune system without overstimulating the immune response (6, 10).

Anti-inflammatory action. Biomodulina T significantly modified the inflammatory response in carrageenan-induced paw edema, a rat model of acute inflammation (6, 11).

Cotton pellet-induced granuloma formation is a model of chronic inflammation in rats. Biomodulina T inhibited chronic inflammation and the release of arachidonic acid from activated macrophages, which was related to an inflammation-modulating effect. Biomodulina T downregulated inflammatory mediators and decreased inflammation (6, 11).

## Clinical Uses of Biomodulina T

The first immunocompromised child to receive Biomodulina T had serious infections due to selective deficiency of IgA. When we administered Biomodulina T, the patient responded considerably, as evidenced by increases in CD3+, CD4+, and CD8+ T-cells, recovery of normal levels of IgA, weight gain, and resolution of serious respiratory and gastrointestinal infections. This was the first clinical report we published on the use of Biomodulina T in immunodeficiency disease as well as the first report of the clinical effect of Biomodulina T-mediated restoration of T cell populations and IgA (6, 12).

Biomodulina T has been used in the treatment of infectious diseases, autoimmune diseases, immunodeficiency, and cancer (6, 12–26). In 1994, we obtained approval for Biomodulina T as an immunomodulatory drug (27).

Clinical trials have shown that Biomodulina T restores CD3+, CD4+, and CD8+ T-cells and their function and enhances the immune response against infections (6, 12–26). Biomodulina T restores immunosenescent CD4+ and CD8+ T-cells in older adults and improves the proliferative capacity of CD4+ T-cells and their ability to produce cytokines (26).

Biomodulina T has an excellent safety profile, has been well tolerated, and has not been associated with any significant side effects and contraindications (6).

## T Cells, COVID-19, and Biomodulina T

Aging and scarcity of naive T-cells may be linked risk factors for severe COVID-19 (28). Reduced ability to respond to new antigens is linked to a decrease in the number of peripheral naïve T and B cells. Naïve T-cells are abundant in youth but may be exhausted by exposure to microorganisms over the course of a lifetime, differentiating into memory lymphocyte subsets (29).

Prominent lymphopenia with a reduction in the number of T-cells is observed in patients with severe COVID-19. T-cells have an important role in COVID-19 and in immunological memory following SARS-CoV-2 infection. T-cells represent a clue for new opportunities for the treatment and prevention of COVID-19 (30).

Patients recovered from COVID-19 have substantial CD4+ and CD8+ T cell responses to SARS-CoV-2. CD4+ T-cells respond strongly to the spike protein, the main target of most vaccines, and correlate with the magnitude of anti-SARS-CoV-2 IgG and IgA titers. Additionally, the CD4+ T-cell response is detected in nonexposed individuals due to cross-reactive T-cell recognition between circulating “common cold” coronaviruses and SARS-CoV-2 (31). The extensive heterogeneity in COVID-19 disease may be explained in part by individual T-cell memory of coronaviruses that cause the common cold (32).

Older adults are most susceptible to developing the most serious manifestations of the disease produced by the SARS-CoV-2 virus due to the aging of the immune system, a phenomenon known as immunosenescence that is related to the inflammaging process of chronic inflammation (33). During aging, changes in innate and adaptive immune responses occur. In the elderly, involution in the thymus gland causes a decrease in the production of thymic factors and thymopoiesis. Thymic involution is one of the major features of human immunosenescence (34). The thymus is the primary lymphoid organ where naïve T-cells are generated, and progressive involution of the thymus, and the decrease in thymopoiesis are the reasons that the number of naïve T-cells declines during aging. T-cells exhibit progressive shrinking of the immune repertoire during aging, with a reduced proportion of naïve cells and an increased proportion of memory and effector cells and pro-inflammatory cytokines. This is the reason older adults have a decreased ability to respond to new antigens and vaccines and activate an immune response in an uncontrolled way that leads to the exaggerated release of cytokines, a phenomenon known as the cytokine storm, which causes damage to cells, tissues, and vital organs. T-cells function as a negative regulator of the innate immune response and calm the cytokine storm (35).

Age-related regression of the thymus, associated with a decline in naïve T-cell output, contributes to the reduction in T-cell diversity in older individuals and is linked with increased susceptibility to infection, autoimmune disease, and cancer. Mortality in COVID-19 increases with age, with the highest mortality among those over 80 years of age (36).

The generation and maintenance of the diverse peripheral naïve T-cell repertoire are critical to the normal function of the immune system (34). In the elderly and in immunocompromised individuals, it is of vital importance to improve the effectiveness of the adaptive immune response, restore T-cells for a better response to virus infection, and generate a better response to future vaccines. At the same time, modulation of the inflammatory process can improve the prognosis of patients with the most serious manifestations of COVID-19.

Coordinated SARS-CoV-2-specific adaptive immune responses are associated with milder disease, suggesting roles for both CD4+ and CD8+ T-cells in protective immunity in COVID-19, and are disrupted in older adults. Scarcity of naive T-cells was also associated with aging and poor disease outcomes. Coordinated CD4+ T-cell, CD8+ T-cell, and antibody responses are protective, but uncoordinated responses fail to control disease, with a connection between aging and impaired adaptive immune responses to SARS-CoV-2 (28).

**Figure 1:**
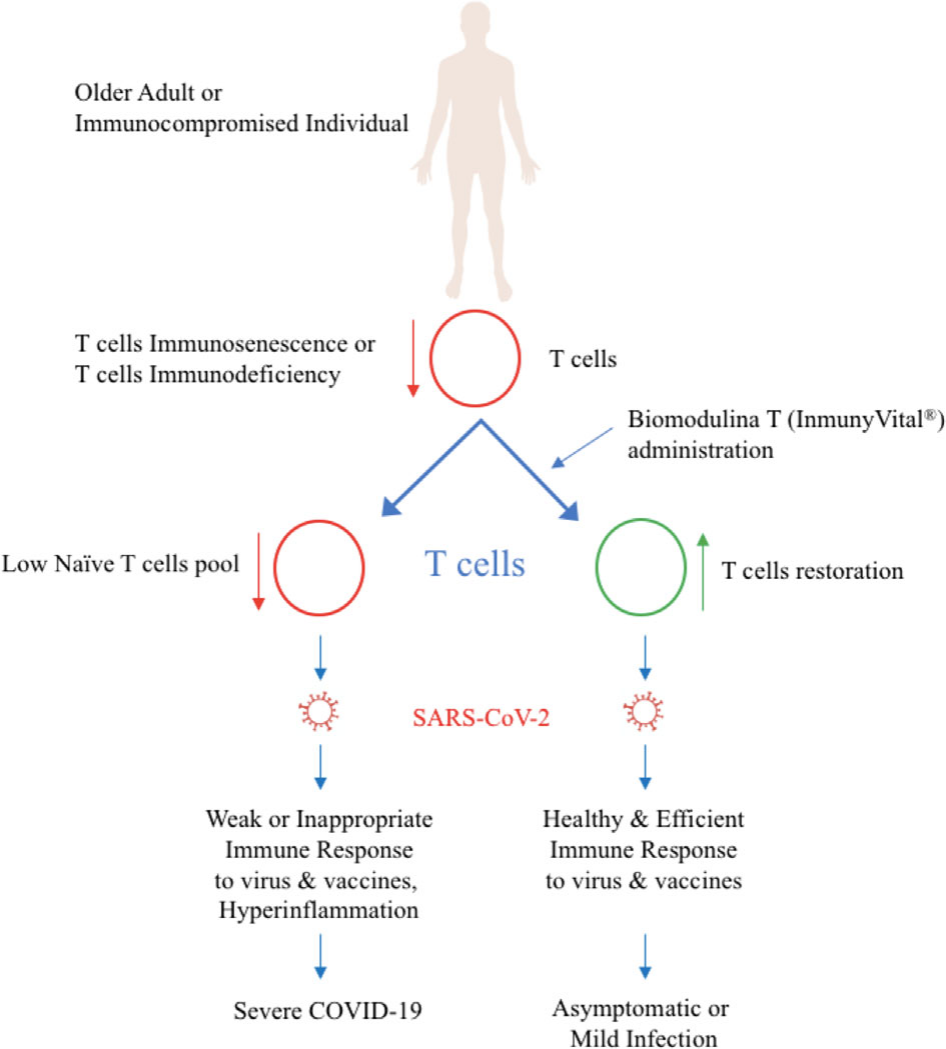
Biomodulina T (InmunyVital®) restores T cells and helps contain COVID-19.

Biomodulina T, the active ingredient in InmunyVital^®^, modulates immune responses and inflammation and helps contain COVID-19 (**Figure 1**). Biomodulina T restores T-cells and immunity in immunocompromised people and in older adults and helps reverse immunosenescence. Clinical trials in immunodeficiency, autoimmune diseases, and infectious diseases show that Biomodulina T restores CD3+, CD4+, and CD8+ T-cells and their function and enhances the immune response against infections (6, 12–26). In older adults, Biomodulina T increases the numbers of CD4+ naïve T-cells, CD4+ recent thymic emigrant (RTE) T-cells, CD8+ stem cell-like memory (SCM) T-cells, and CD4+ CD31+ naïve T-cells without changes in the frequency of regulatory T lymphocytes. Biomodulina T also improves the proliferative capacity of CD4+ T-cells and the ability of CD4+ T-cells to produce IFN-γ and helps restore the Th1 response (26). Biomodulina T leads to restoration of the normal thymus environment, increasing the number of naïve T-cell repertoires and memory T-cells with the characteristics of stem cells and the capacity for self-renewal and proliferation. The expression of programmed death 1 (PD1), a marker of T-cells exhaustion decreased in CD4+ and CD8+ T-cells after the administration of Biomodulina T, this suggest an anti-exhaustion effect (26, 37). All these factors contribute to restoration of a healthy immune response and to immune system homeostasis.

Biomodulina T is currently being used in a phase IV clinical trial to prevent COVID-19 (38). Biomodulina T is being administered in more than 10,000 older adults during this COVID-19 pandemic, mainly to SARS-CoV-2-positive patients and to other people in nursing homes as a preventive measure, considering that they are at increased risk of having the most serious manifestations of the disease.

The goal of treatment is to improve the immune response and health in older adults and help contain the transmission of COVID-19 and other infectious diseases in nursing homes.

Biomodulina T (InmunyVital^®^) is an alternative for the prevention and treatment of COVID-19. The biological effects of Biomodulina T and the clinical results obtained in immune disorders suggest that the use of Biomodulina T is a promising strategy to reverse T-cell immunosenescence and restore the immune response in older adults and immunocompromised people, decreasing the risk of more serious complications from COVID-19.

Health measures taken as well as the application of vaccines in development will soon help contain COVID-19, but beyond that, new and universal prevention strategies are needed.

Biomodulina T (InmunyVital^®^) modulates the immune response and inflammation, restores immunity, and can become a global strategy to prevent, treat, and contain COVID-19 and other current and future infectious diseases. It can also be used as a vaccine enhancer and immunotherapy agent to support the treatment of cancer and other immune disorders and improve the healthspan.

## Author Contributions 

All authors contributed to the article and approved the submitted version.

## Conflict of Interest

The authors are affiliated with BIOLONG. RRRM is the discoverer and lead developer of Biomodulina T (InmunyVital^®^), the Founder and President of BIOLONG, the company commercializing InmunyVital^®^ (Biomodulina T).

## References

[1] Rodriguez Martin RR Vega Sanchez H Pelaez Borges A Biomoduladores Tímicos. Revisión Bibliográfica. Rev Cubana Farmacia (1988) 22(2):72–83.

[2] Rodriguez Martin RR Tratan hepatitis y esclerosis múltiple con sustancia extraída del timo bovino. Periódico Granma (1989) 25(243):1–2.

[3] Rodriguez Martin RR Gonzalez Ross JE Gonzalez Gonzalez O Pelaez Borges A Caracterización fisicoquímica de una fracción de timo. I Aspectos generales. Rev Cubana Med Militar (1993) 22(1):17–23.

[4] Rodriguez Martin RR Biomodulina T. Consulta Médica. Periódico Granma. Junio 1. La Habana, Cuba: Granma (1996).

[5] Rodriguez Martin RR Biomodulina T, Múltiples usos. Rev. Avances Médicos de Cuba. Año IV No. 9. La Habana, Cuba: Prensa Latina (1997). pp. 60–2.

[6] Rodriguez Martin RR History of the Discovery and Development of Biomodulina T (InmunyVital®), a Useful Immunomodulator with a Broad Range of Clinical Applications. Available at: https://ssrn.com/abstract=3678181 10.2139/ssrn.3678181 (Accessed July 2, 2020). 10.2139/ssrn.3678181 37086680

[7] Exley AR Buckenham S Hodges E Hallam R Byrd P Last J Premature aging of the Immune System underlies immunodeficiency in Ataxia-Telangiectasia. J Clin Immunol (2011) 140:26–36. 10.1016/j.clim.2011.03.007 21459046

[8] Rodriguez Martin RR Gonzalez Gonzalez O Ensayo de Actividad Biológica de una fracción de Timo: Biomodulina T. Rev Cubana Med Militar (1993) 22(2):108–11.

[9] Wilson AB Coombs RRA Rosette-formation between guinea pig lymphoid cells and rabbit erythrocytes – a possible T-cell marker. Int Arch Allergy (1973) 44:544–52. 10.1159/000230959 4123914

[10] Rios Hernandez M Formell J Cruz M Rodriguez Martin RR Calzada S Bello JL Evaluación de la actividad biológica y aspectos inmunofarmacológicos de la Biomodulina T. Documentación Registro Sanitario CECMED. La Habana, Cuba: Departamento de Investigaciones Preclínicas. Grupo de Farmacologia, Instituto Nacional de Oncologia y Radiologia, INOR (1993).

[11] Leon Cofiño L Valdes Rodriguez Y Rios Hernandez M Estudio de la acción antiinflamatoria de la Biomodulina T. Tesis de grado. Lic. en Farmacia. Facultad de Farmacia y Alimentos. La Habana, Cuba: Universidad de La Habana (1994).

[12] Rodriguez Martin RR Vega Sanchez H Gonzalez Gonzalez O Pelaez Borges A Tratamiento de la Inmunodeficiencia Selectiva de IgA con Biomodulina T. Rev Cubana Med Militar (1994) 23(1):31–5.

[13] Vega Sanchez H Rodriguez Martin RR Gonzalez Gonzalez O Pelaez Borges A Enfermedad de Behcet. Tratamiento con Biomodulina T. Rev. Cub. Inv. Biomed. Número Especial. La Habana, Cuba: XVII Congreso Asoc. Lat. Ciencias Fisiol. (1991).

[14] Jimenez Mesa G Vega Sanchez H Rodriguez Martin RR Collazo Borrego L Castellanos O Gra Oramas B Tratamiento de la Enfermedad de Crohn con Biomodulina T. XXII Congreso Panamericano de Enfermedades Digestivas. 14-18 octubre. La Habana: Organizacion Panamericana Gastroenterologia (1991).

[15] Devesa Colina E Garcia Lara R Rodriguez Martin RR Rodriguez Nuñez OM Alonso Abad A Pascual Lopez MA La Biomodulina T como inmunomodulador en geriatría. Ensayo clínico controlado, fase II. La Habana, Cuba: Centro Nacional Coordinador de Ensayos Clínicos (1994).

[16] Chiong Wong R Campillo Motilva R Rodriguez Martin RR Gonzalez Gonzalez O Pelaez A Gonzalez Ross JE Tratamiento de la Artritis reumatoide y Artritis reactiva con Biomodulina T. Documentación Registro Sanitario. La Habana, Cuba: CECMED (1994).

[17] Vega Sanchez H Llanio Navarro R Rodriguez Martin RR Gra Oramas B Collazo Borrego L Apesteguia Garcia S Tratamiento de Hepatitis Crónica Activa tipo B con Biomodulina T combinado con interferón alfa leucocitario. Documentación Registro Sanitario Biomodulina T. La Habana, Cuba: CECMED (1993).

[18] Christian Lopez L Rodriguez Martin RR Rabassa J Santamaria Lafargue M Romero del Sol JM Gonzalez Ross JE Efecto de la Biomodulina T 1000 sobre el Timo en niños con infecciones recurrentes. Rev Cubana Pediatría (2000) 72(1):3–9.

[19] Molinet Fuertes EJ Moulton Alvarez J Gonzalez Sirut J Muñoz B Leyva B Biomodulina T, una opción terapéutica. Rev Cubana Reumatología (2002) Volumen IV:68.

[20] Lara Rodriguez RF Rodriguez Martin RR Vargas Batista A Bolet Arostavista M Fernandez Carriera RA Suarez Luis I Tratamiento de la exacerbación en Esclerosis Múltiple con Biomodulina T. Informe final. Ensayo clínico controlado, fase II; CENCEC. La Habana, Cuba: CECMED (2003).

[21] Gamez Morales LA Lara Rodriguez RF Rodriguez Martin RR Gonzalez-Quevedo Monteagudo A Fernandez Carreira R Marzoa Silva N Estudio fase II de tratamiento de pacientes con Esclerosis múltiple exacerbante-remitente con Biomodulina T. Rev Mex Neurociencias (2007) 8(1):28–31.

[22] Jauma AJ Insua C Macias C Padilla M Gonzalez C Uso de la Biomoduina T en el Síndrome de Di George. Presentación de un caso. VacciMonitor (2011) 20 Supplement:1.

[23] Marsan Suarez V Garcia A De Leon N Macias C Sanchez M Benitez D Síndrome de Edwards asociado a inmunodeficiencia combinada. Rev Cubana Hematol Inmunol Hemoter (2011) 27(3):342–8.

[24] Garcia M Suarez R Castro I Santiago D Alfonso I Efecto terapéutico de la Biomodulina T en pacientes portadores de enfermedad pulmonar obstructiva crónica. Rev Habanera Cienc Med (2011) 10(3):287–95.

[25] Garcia M Capdevilla V Suarez R Rodriguez L Castro I Efecto de la Biomodulina T sobre las infecciones respiratorias altas y la polifarmacia en el anciano. Rev Habanera Cienc Med (2014) 13(3):425–36.

[26] Saavedra D Fuertes SA Suarez GM Gonzalez A Lorenzo-Luaces P Garcia B Biomodulina T partially restores immunosenescent CD4 and CD8 T cell compartments in the elderly. Exp Gerontol (2019) 124:110633. 10.1016/j.exger.2019.110633 31207285

[27] Rodriguez Martin RR Registro Sanitario de Biomodulina T. Centro Estatal para el Control de la Calidad de los Medicamentos (CECMED). La Habana, Cuba: Ministerio de Salud Pública de Cuba (1994).

[28] Moderbacher CR Ramirez SI Dan JM Grifoni A Hastie KM Weiskopf D Antigen-specific Adaptive Immunity to SARS-CoV-2 in Acute COVID-19 and Associations with Age and Disease Severity. Cell (2020) 183(4):996–1012. 10.1016/j.cell.2020.09.038 33010815 PMC7494270

[29] Aiello A Farzaneh F Candore G Caruso C Davinelli S Gambino CM Immunosenescence and Its Hallmarks: How to Oppose Aging Strategically? A Review of Potential Options for Therapeutic Intervention. Front Immunol (2019) 10:2247. 10.3389/fimmu.2019.02247 31608061 PMC6773825

[30] Zheng HY Zhang M Yang CX Zhang N Wang XC Yang XP Elevated exhaustion levels and reduced functional diversity of T cells in peripheral blood may predict severe progression in COVID-19 patients. Nature Cell Mol Immunol (2020) 17:541–43. 10.1038/s41423-020-0401-3 PMC7091621 32203186

[31] Grifoni A Weiskopf D Ramirez SI Mateus J Dan JM Moderbacher CR Targets of T Cell Responses to SARS-CoV-2 Coronavirus in Humans with COVID-19 Disease and Unexposed Individuals. Cell (2020) 181(7):1489–501.e15. 10.1016/j.cell.2020.05.015 PMC7237901 32473127

[32] Mateus J Grifoni A Tarke A Sidney J Ramirez SI Dan JM Selective and cross-reactive SARS-CoV-2 T cell epitopes in unexposed humans. Science (2020) 370(6512):89–94. 10.1126/science.abd3871 32753554 PMC7574914

[33] Fulop T Larbi A Dupuis G Le Page A Frost EH Cohen AA Immunosenescence and inflamm-aging as two sides of the same coin: Friends or foes? Front Immunol (2018) 8:1960. 10.3389/fimmu.2017.01960 29375577 PMC5767595

[34] Lang PO Govind S Aspinall R Reversing T cell immunosenescence: why, who, and how. Age (Dordr) (2013) 35(3):609–20. 10.1007/s11357-012-9393-y PMC3636382 22367580

[35] Tufet M T cells calm the storm. Nat Rev Immunol (2007) 7:834–5. 10.1038/nri2197

[36] Centers for Disease Control and Prevention (CDC) People who are at higher risk for severe illness. United States: Centers for Disease Control and Prevention (CDC) (2020). CDC.gov/coronavirus/2019-ncov/2020.

[37] Suarez-Formigo GM Saavedra-Hernandez D Biomodulina T May Restore Immunity in Older Adults. MEDICC Rev (2020) 22(3):54–6. 10.37757/MR2020.V22.N3.11 32812900

[38] Use of Biomodulina T for the prevention of infections, including COVID-19 in older adults. Phase IV clinical trial. International Clinical Trials Registry Platform. Geneva, Switzerland: World Health Organization (2020).

